# Case Report: Acute Pancreatitis Associated With Tacrolimus in Kidney Transplantation and a Review of the Literature

**DOI:** 10.3389/fmed.2022.843870

**Published:** 2022-04-21

**Authors:** Yixuan Ding, Chang Qu, Huan He, Feng Cao, Tongwen Ou, Fei Li

**Affiliations:** ^1^Department of General Surgery, Xuanwu Hospital, Capital Medical University, Beijing, China; ^2^Clinical Center for Acute Pancreatitis, Capital Medical University, Beijing, China; ^3^Department of Urology, Xuanwu Hospital, Capital Medical University, Beijing, China; ^4^Department of Urology, Xuanwu Hospital, Capital Medical University, Beijing, China

**Keywords:** drug-induced pancreatitis, tacrolimus, kidney transplantation, acute pancreatitis, abdominal pain

## Abstract

**Background:**

Drug-induced pancreatitis is a rare cause of acute pancreatitis. Tacrolimus has been used as an immunosuppressant agent in patients after organ transplantation. However, only a few case reports of tacrolimus-induced acute pancreatitis in kidney transplantation have been reported. The purpose of this case report is to alert clinicians that tacrolimus-induced acute pancreatitis may occur during tacrolimus therapy in kidney transplant patients.

**Case Presentation:**

We present the case of a 38-year-old woman who underwent kidney transplantation and received immunosuppressive therapy with tacrolimus; on day 20 post-transplantation, she presented with acute abdominal pain in the middle and left areas of the abdomen accompanied by diarrhea, nausea, and vomiting. We excluded gallstone disease, alcohol, hypertriglyceridemia, and other possible causes, and speculated that tacrolimus was the probable cause of pancreatitis because of the extremely high blood concentration of tacrolimus. After tacrolimus was changed to cyclosporine, her symptoms were gradually improved, and she was discharged home without relapse.

**Conclusion:**

Tacrolimus is a rare cause of pancreatitis after kidney transplantation. It is important to note that tacrolimus-induced acute pancreatitis may occur during tacrolimus therapy in kidney transplantation patients.

## Introduction

Drug-induced pancreatitis (DIAP) is considered a rare cause of acute pancreatitis (AP) and accounts for ~2–5% of all AP cases (1, 2). The World Health Organization database has indicated that more than 500 drugs may cause DIAP, and at least 30 drugs have been directly associated with the etiology of AP (3, 4). Therefore, excluding gallstone disease, alcohol, hypertriglyceridemia, and other possible causes, the administration of associated drugs may lead to the occurrence of AP.

The common causative drugs of DIAP hospitalization are azathioprine, atorvastatin, and hydrochlorothiazide (5). Tacrolimus has been used as an immunosuppressant agent in patients who have undergone organ transplantation. However, only a few case reports of tacrolimus-induced AP after kidney transplantation have been reported (6, 7). Here, we report a case of tacrolimus-induced AP after kidney transplantation and review the relevant literature.

## Case Presentation

A 38-year-old female with uremic stage of chronic renal insufficiency received a kidney transplant from cardiac death donor. She had been diagnosed with chronic renal insufficiency 14 years previously. The patient was treated with peritoneal dialysis for 4 months. She had no history of gallstones, alcohol, or hypertriglyceridemia. Her body mass index was 17.9 kg/m^2^ (height, 153 cm; weight, 42 kg). She underwent a kidney transplant at our institution in March 2021. Before the surgery, she received antilymphocyte therapy with basiliximab (20 mg intravenously) and corticosteroids (10 mg/kg). The surgical procedure was successful, and the initial immunosuppressive regimen consisted of tacrolimus (4 mg/day), enteric-coated mycophenolate sodium (1,500 mg/day), and corticosteroids (35 mg/day). Meropenem (1 g every 8 h) and caspofungin (50 mg/day) were used to prevent post-operative infection.

**Figure 1:**
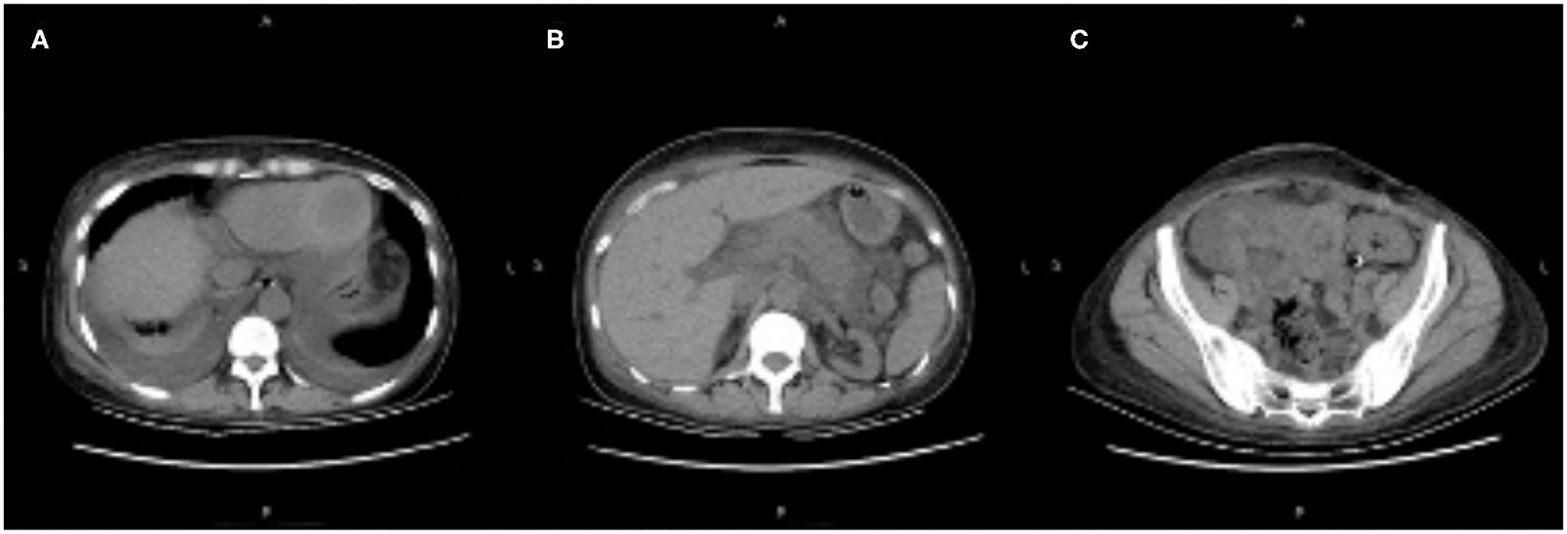
**(A–C)** Abdominal computed tomography scan showing pancreatic enlargement with peripancreatic inflammatory exudate, bilateral pleural effusion, and the transplanted kidney.

On day 20 post-transplantation, she presented with acute abdominal pain in the middle and left abdominal areas accompanied by diarrhea, nausea, and vomiting. Physical examination revealed moderate abdominal tenderness. Laboratory analysis showed white blood cells 9.12 × 10^9^/L, neutrophils 8.32 × 10^9^/L, hemoglobin 75 g/L, platelets 396 × 10^9^/ L, blood creatinine 272 μmol/L, blood urea nitrogen 38.5 mmol/L, triglycerides 0.64 mmol/L, serum amylase 450 IU/L (normal 15–115), lipase 345 U/L (normal 6–51), and trough concentration tacrolimus >30 ng/ml (dosage was 6 mg/day). On day 21, her abdominal pain worsened, and physical examination revealed moderate abdominal tenderness and rebound tenderness. An abdominal computed tomography (CT) scan showed pancreatic enlargement with peripancreatic inflammatory exudate, bilateral pleural effusion, and a transplanted kidney (Figure 1). Based on these factors, the patient was diagnosed with pancreatitis.

**Figure 2:**
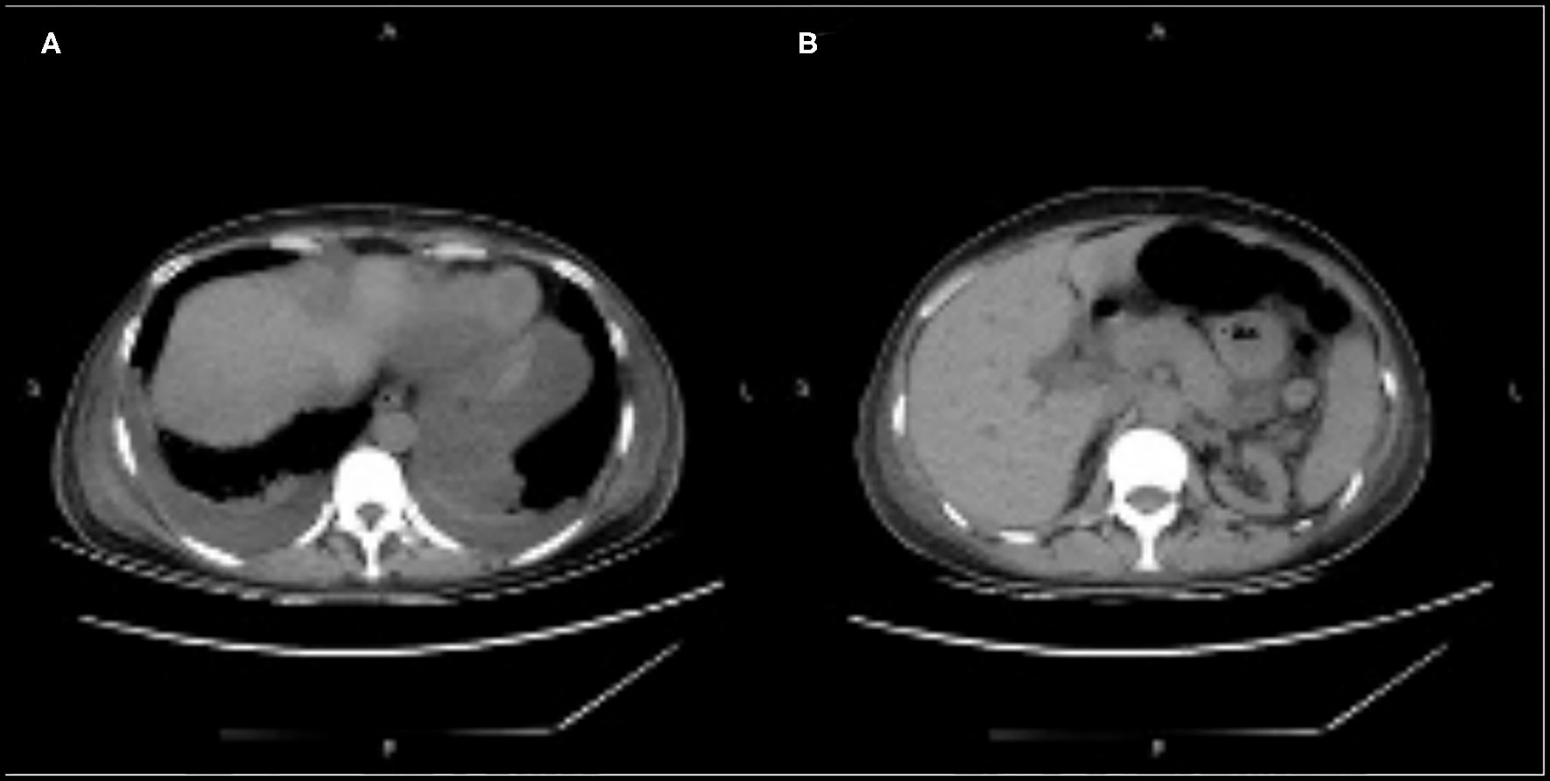
**(A,B)** Computed tomography showing decreased bilateral pleural effusion and peripancreatic effusion.

On day 22, the patient was switched from oral tacrolimus to cyclosporine for injection. Somatostatin was administered to treat AP, and other treatments included fluid replacement, analgesics, and early oral feeding. Fifteen days after treatment (day 37 post-transplantation), the patient's condition improved significantly, her abdominal symptoms were relieved, her blood amylase and lipase levels normalized, and CT showed decreased bilateral pleural effusion and peripancreatic effusion (Figure 2).

**Figure 3:**
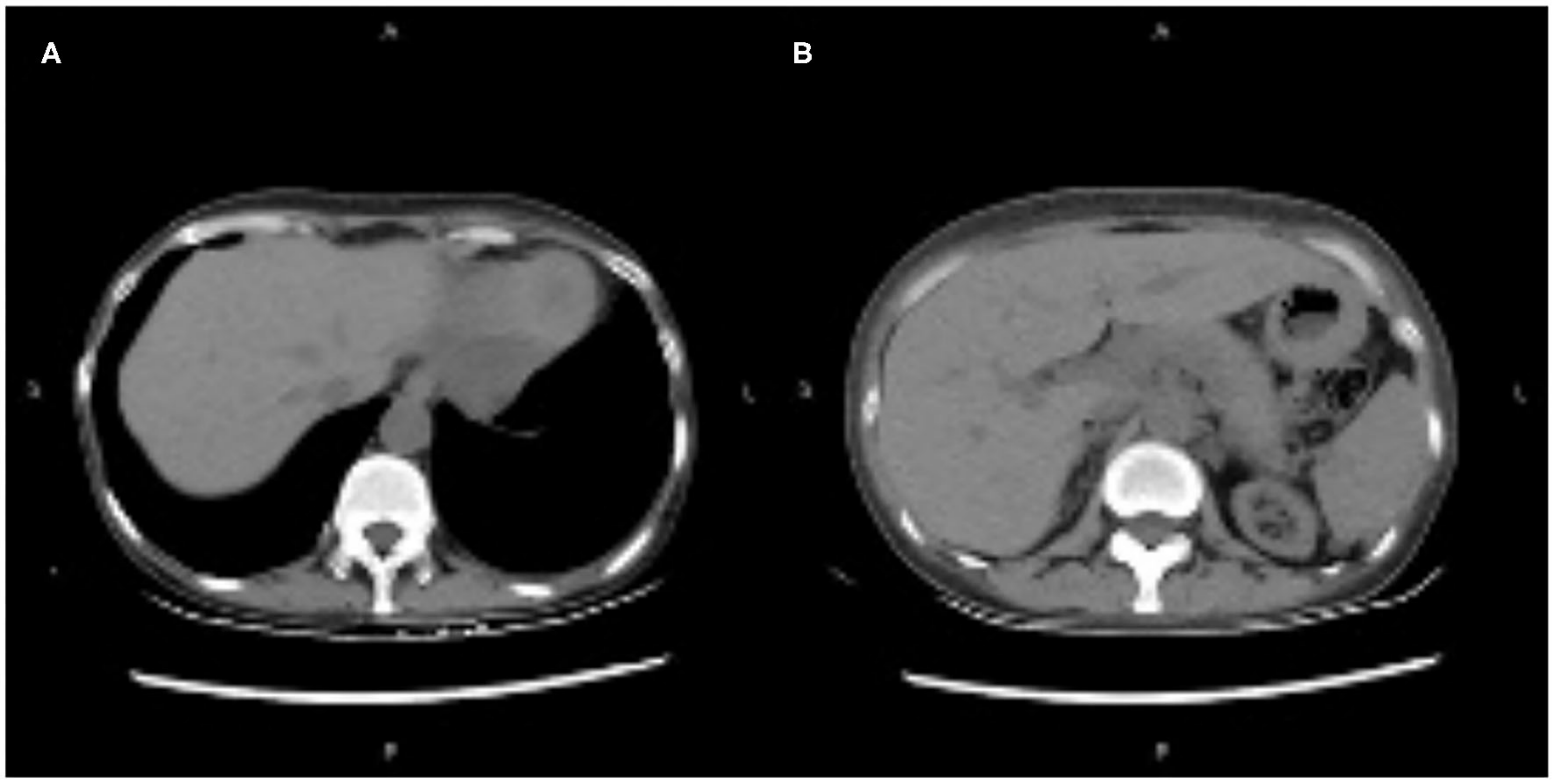
**(A,B)** Computed tomography showing no effusion in the bilateral pleural cavity, and no exudation around peripancreatic and other areas of the abdominal cavity.

**Figure 4:**
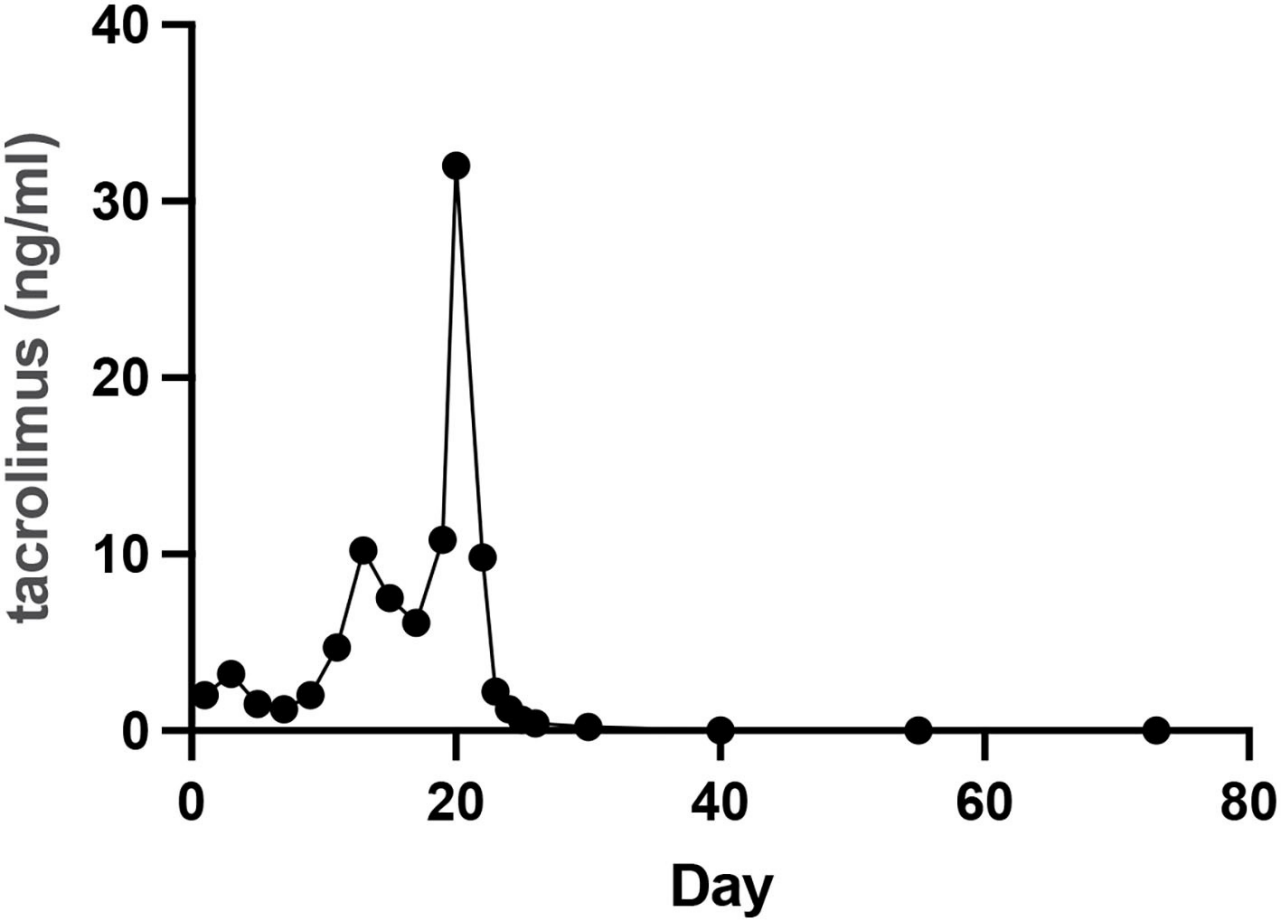
The concentration curve of tacrolimus during the follow-up.

The patient was discharged on day 73. On day 109, CT showed no effusion in the bilateral pleural cavity and no exudation around the peripancreatic or other areas of the abdominal cavity (Figure 3). There was no recurrence of pancreatitis at the 7 months follow-up. Figure 4 showed the concentration curve of tacrolimus during the follow-up.

## Discussion

AP is one of the most common gastrointestinal diseases, with an annual incidence of 110–140 per 100,000 person-year (8). There are no unique clinical features that may distinguish DIAP from other etiologies. Therefore, DIAP can be diagnosed during the drug administration and the AP onset, if all other causes are excluded. Mallory and Kern established a diagnostic criteria for DIAP, Which includes the following: (1) symptoms appear after the administration of the associated drugs, (2) symptoms disappear after drug withdrawal, (3) other causes are excluded, and (4) symptoms recur after rechallenge (9). DIAP drugs can be classified into four classes (class I-IV) based on the latency period, number of cases, reaction with rechallenge, and the exclusion of other AP causes (3). Class Ia drugs that included medications with at least one case report with positive rechallenge and other causes such as gallstones, alcohol, and hypertriglyceridemia were excluded. Class Ib drugs, including drugs with at least one case, have been reported with a positive rechallenge, but other AP causes cannot be excluded. Class II drugs including drugs with at least four cases have been reported, and there is a coincident latency in more than 75% of cases. Class III drugs included at least two cases in the literature with no consistent latency period between cases, and no rechallenge. Class IV drugs have not been reported previously, with a single case reported and without rechallenge (10). According to this classification, tacrolimus is considered to be the cause of AP among the drugs the patient was taking at the time of AP onset. The present case is the third reported case of AP caused by tacrolimus after renal transplantation and meets the diagnostic criteria (Table 1).

**Table 1:** Summary of cases of acute pancreatitis due to tacrolimus in kidney transplantation.

**References**	**Age**	**Gender**	**Diagnosis time**	**Concentration of tacrolimus**	**Treatment**	**Follow-up**
Xu et al. (6)	45	Male	On day 67	>30 ng/ml	Change to cyclosporine	7 months
Liu et al. (7)	24	Male	On day 10	>30 ng/ml	Change to cyclosporine	4 months
Ding et al. (this study)	38	Female	On day 20	>30 ng/ml	Change to cyclosporine	7 months

In our report, at the time of onset of pancreatitis, the patient received various drugs, including tacrolimus, enteric-coated mycophenolate sodium, corticosteroids, meropenem, caspofungin, and omeprazole. Tacrolimus, corticosteroids, meropenem and omeprazole may cause AP. However, the concentration of tacrolimus was >30 ng/ml; moreover, pancreatitis was relieved and did not relapse when tacrolimus was stopped. Based on the classification criteria, we can consider a possible association between tacrolimus and pancreatitis. When tacrolimus was changed to cyclosporine, the patient's condition improved significantly, her abdominal symptoms were relieved, her blood amylase and lipase levels normalized, and her CT showed decreased bilateral pleural effusion and peripancreatic effusion.

Although tacrolimus has been widely used in transplantation, AP induced by tacrolimus has rarely been reported. A review of the relevant literature showed that only two reports have reported AP associated with tacrolimus after kidney transplantation (6, 7). Mechanisms of tacrolimus-induced AP may include immunologic reactions, cell metabolism, and systemic or local infection. Other tacrolimus-induced AP causes include heart, liver, and lung transplantation (11–13). According to Mallory and Kern's diagnostic criterion for DIAP, our case met the first three criteria (9). After reviewing the literature, this is the third reported case of pancreatitis caused by tacrolimus in kidney transplantation. Based on the experience of the first two reports, tacrolimus was not administered to this patient. This case excluded gallstone disease, alcohol, hypertriglyceridemia, and other possible causes; at the same time, the administration of tacrolimus lead to the occurrence of AP. Therefore, we consider this case to be the most probable association between pancreatitis and tacrolimus.

## Conclusion

After reviewing the literature, this is the third reported case of pancreatitis caused by tacrolimus in kidney transplantation. It is very rare that AP is an adverse effect of tacrolimus; therefore, clinicians should alert other clinicians that tacrolimus-induced AP may occur during tacrolimus therapy in kidney transplantation patients. Pancreatitis should be promptly suspected in every patient who develops acute abdominal pain after kidney transplantation.

## Data Availability Statement

The original contributions presented in the study are included in the article/supplementary material, further inquiries can be directed to the corresponding authors.

## Ethics Statement

The studies involving human participants were reviewed and approved by Bioethics Committee of Xuan Wu Hospital, Capital Medical University. The patients/participants provided their written informed consent to participate in this study.

## Author Contributions

All authors listed have made a substantial, direct, and intellectual contribution to the work and approved it for publication.

## Funding

This study supported by Beijing Municipal Science and Technology Commission (Z171100001017077), Beijing Municipal Science and Technology Commission Clinical Diagnosis and Treatment Technology Research and Demonstration Application Project (Z191100006619038), Capital Medical Development and Research Special Project (2020-1-2012 and Z201100005520090), and Construction Project of Clinical Advanced subjects of Capital Medical University (1192070312).

## Conflict of Interest

The authors declare that the research was conducted in the absence of any commercial or financial relationships that could be construed as a potential conflict of interest.

## Publisher's Note

All claims expressed in this article are solely those of the authors and do not necessarily represent those of their affiliated organizations, or those of the publisher, the editors and the reviewers. Any product that may be evaluated in this article, or claim that may be made by its manufacturer, is not guaranteed or endorsed by the publisher.
